# The Insulin Receptor Mediates Insulin’s Early Plasma Clearance by Liver, Muscle, and Kidney

**DOI:** 10.3390/biomedicines9010037

**Published:** 2021-01-05

**Authors:** Rick I. Meijer, Eugene J. Barrett

**Affiliations:** Division of Endocrinology, Department of Medicine, University of Virginia Health Care System, Charlottesville, VA 22908, USA; r.meijer@amsterdamumc.nl

**Keywords:** insulin clearance, insulin receptor, muscle, liver, kidney, S961

## Abstract

The role of the insulin receptor in mediating tissue-specific insulin clearance in vivo has not been reported. Using physiologic insulin doses, we measured the initial clearance rate (first 5 min) of intravenously injected ([^125^I]Tyr^A14^)-insulin by muscle, liver, and kidney in healthy rats in the presence and absence of the insulin receptor blocker S961. We also tested whether 4 weeks of high-fat diet (HFD) affected the initial rate of insulin clearance. Pre-treatment with S961 for 60 min prior to administering labeled insulin raised plasma ([^125^I]Tyr^A14^)insulin concentration approximately 5-fold (*p* < 0.001), demonstrating receptor dependency for plasma insulin clearance. Uptake by muscle (*p* < 0.01), liver (*p* < 0.05), and kidney (*p* < 0.001) were each inhibited by receptor blockade, undoubtedly contributing to the reduced plasma clearance. The initial plasma insulin clearance was not significantly affected by HFD, nor was muscle-specific clearance. However, HFD modestly decreased liver clearance (*p* = 0.056) while increasing renal clearance by >50% (*p* < 0.01), suggesting a significant role for renal insulin clearance in limiting the hyperinsulinemia that accompanies HFD. We conclude that the insulin receptor is a major mediator of initial insulin clearance from plasma and for its clearance by liver, kidney, and muscle. HFD feeding increases renal insulin clearance to limit systemic hyperinsulinemia.

## 1. Introduction

Insulin activates signaling networks in many tissues by binding to the insulin receptor (IR) [1]. Insulin’s action in the liver increases IR endocytosis and insulin clearance [2]. Kidney, muscle, adipose, brain, and other tissues also clear insulin [3,4]. The IR’s role in systemic insulin clearance at these other sites has not been defined. We recently reported that S961 (a specific IR blocker), inhibits ([^125^I]Tyr ^A14^)insulin’s initial brain clearance [5] by blocking insulin uptake by the brain microvascular endothelial cell [6], implicating vascular IRs in brain insulin clearance at physiological insulin concentrations. Unlabeled insulin, in vivo, inhibits skeletal muscle uptake of labeled insulin, indicating that insulin uptake is a saturable process [7], perhaps involving IR or insulin-like growth factor-1 (IGF-1) receptors (IGF-1R) or IR/IGF-1R hybrid receptors. Here, we used S961 (which specifically blocks IR, not IGF-1R or hybrid receptors) to test IR’s role in whole-body (plasma) and tissue-specific (skeletal muscle, liver, and kidney) insulin clearance in rats. We also tested the effect of 4 weeks of high-fat diet (HFD) feeding on systemic and tissue-specific insulin clearance. HFD [8] and increased liver fat [9] inhibit liver insulin clearance, and its effect on insulin’s clearance by other tissues has, to our knowledge, not been reported.

## 2. Materials and Methods

### 2.1. Animal Study Protocol

Two groups of male Sprague-Dawley rats were studied. Group 1 rats (*n* = 20, weighing 275 ± 8 g) were fed a normal chow diet; group 2 rats (*n* = 14) were fed either a HFD (60% of calories from fat, *n* = 7, weight 444 ± 8 g) or chow (*n* = 7, weight 400 ± 6 g) for four weeks. The study protocols were approved by the University of Virginia Institutional Animal Care and Use Committee (protocol No. 4104, approved 14 November 2017). Overnight fasted rats were anesthetized with thiobutabarbital (180 mg/kg); both jugular veins were cannulated and used for either ([^125^I]TyrA14)-insulin infusion or blood sampling. Rats were allowed to stabilize for 30 min after surgery before beginning the infusion study. Ten rats from group 1 were given 20 nmol/kg S961 intravenously 60 min before insulin administration [10], while control rats received only saline. S961 was generously provided by Novo-Nordisk, Copenhagen, Denmark.

### 2.2. ([125. I]TyrA14) Insulin Administration and Analysis

A 0.7 pmol ([^125^I]Tyr^A14^) insulin (PerkinElmer, Waltham, MA, USA) bolus was delivered through the right jugular cannula, and subsequently, blood (0.1 mL/min) was withdrawn every min through the left jugular cannula over 5 min. With the rat deeply anesthetized, the left jugular vein was severed and the rat was perfused with ice-cold saline (5 mL/min for 12 min). Skeletal muscle (quadriceps), kidney, and liver were quickly excised and frozen in liquid nitrogen. Blood and tissue samples were powdered, weighed, and mixed with 30% trichloroacetic acid (TCA) and centrifuged. After removing the supernatant, radioactivity in the tissue extracts was measured using a gamma counter (Packard, Cobra II, Canberra, Australia). TCA precipitation of radiolabeled insulin yields estimates of intact insulin comparable to that obtained by immune precipitation [11] but slightly greater than that reported by HPLC analysis [12]. However, given the short study time, and estimates that recirculating radiolabeled insulin degradation products contribute <10% [12] of the total circulating radioactive species during the first 5 min following tracer injection, we opted to use the straightforward TCA precipitation method.

### 2.3. Statistical Analysis

For each study group, comparisons between treatments for plasma insulin clearance and for tissue-specific clearance were made using unpaired Student t-tests. A *p*-value less than 0.05 was considered significant.

## 3. Results

**Figure 1:**
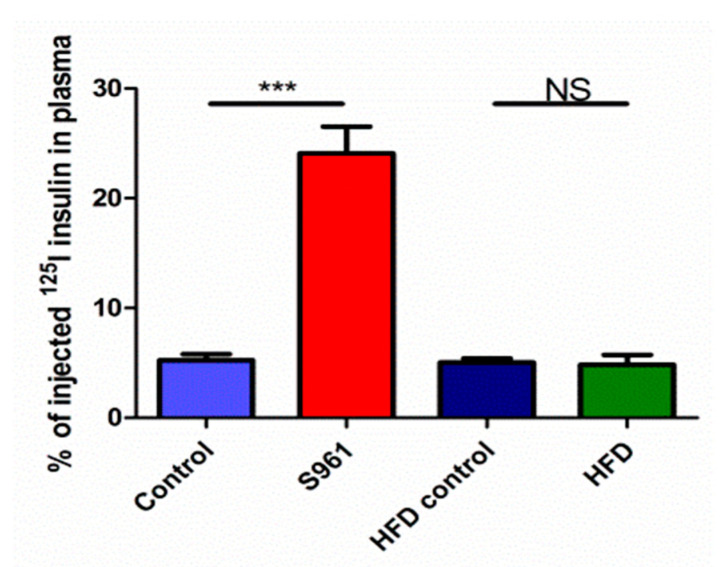
Plasma content of 125 iodine remaining in plasma at five minutes after injection in animal studied under protocol 1 (left two bars) and protocol 2 (right two bars). *** Indicates *p* value less than 0.001 unpaired t-test.

In group 1, at 5 min, only 5.2 ± 0.6% of the labeled insulin remained in the plasma compartment in the control rats (estimated from the product of the measured plasma [^125^I]Tyr^A14^-insulin at 5 min and estimated plasma volume divided by the injected dose) (Figure 1). Compared to the controls, plasma radioisotope retention was greater in S961-treated animals (24.1 ± 2.4% at 5 min, *p* < 0.001), indicating that IR blockade lowers whole-body insulin clearance. The doses of [^125^I]Tyr^A14^-insulin given here (0.7 pmol) to 275–450 g rats, distributes to a plasma volume of ~10–20 mL, which would instantaneously augment plasma insulin concentrations by ~35–70 pM, indicating that the initial insulin clearance rates reported here reflect physiologic insulin concentrations. In contrast to the marked effect of S961, for HFD-fed rats, plasma ^[125I]^TyrA14-insulin retention was 4.8 ± 0.9% and comparable to 5.0 ± 0.4% in age-matched chow-fed controls, *p* = 0.84. Thus, in these Sprague-Dawley rats, 4 weeks of HFD did not affect whole-body insulin clearance.

**Figure 2:**
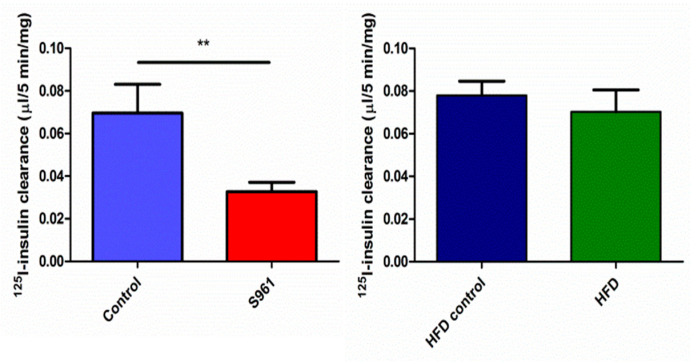
Clearance of radiolabeled insulin by skeletal muscle in rats studied +/−S961 under protocol 1 (left panel) or after feeding a chow diet (blue) or a high-fat diet (green) for four weeks (right panel). ** indicates significance (*p* < 0.01).

**Figure 3:**
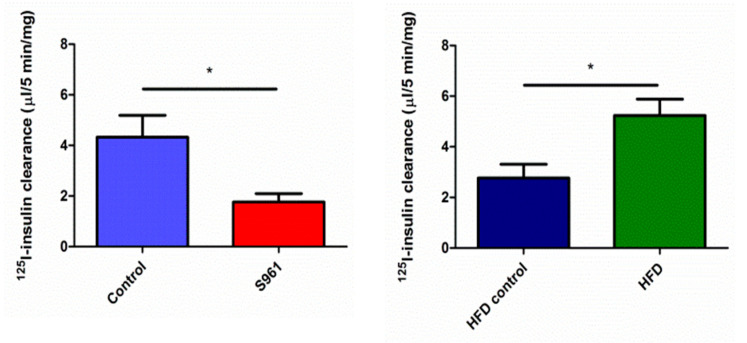
Kidney radiolabeled insulin clearance in rats studied under protocol 1 (left panel) or protocol 2 (right panel). S961 significantly decreased kidney radiolabeled insulin uptake (*; *p* < 0.002). By contrast, high-fat diet significantly increased insulin clearance (*p* < 0.002).

**Figure 4:**
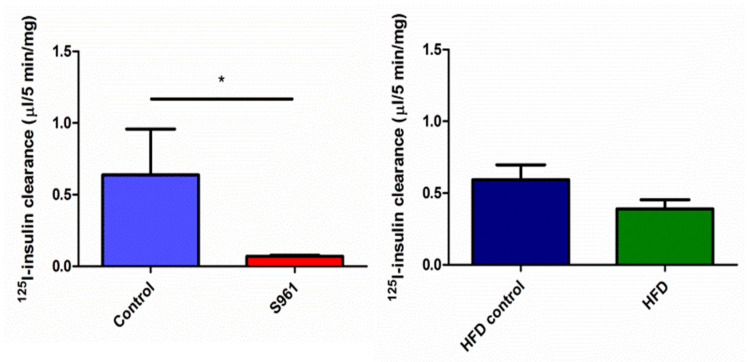
Liver radiolabeled insulin clearance in rats studied under protocol 1 (left panel) or protocol 2 (right panel). S961 significantly decreased radiolabeled insulin uptake (*; *p* = 0.01), while high-fat diet provoked a downward trend in hepatic insulin clearance (*p* = 0.056). Liver radiolabeled insulin clearance in rats studied under protocol 1 (left panel) or protocol 2 (right panel). S961 significantly decreased radiolabeled insulin uptake, and high-fat diet provoked a downward trend in hepatic insulin clearance (*p* = 0.056).

In control rats in group 1, insulin was cleared from plasma by muscle at a rate of 0.070 ± 0.013 µL (5 min)^−1^ mg^−1^, which decreased to 0.03 ± 0.004 µL/5 min/mg after S961 (*p* = 0.002) (Figure 2). Renal insulin clearance/mg tissue was much higher than either muscle or liver (*p* < 0.001, each comparison), and was inhibited by S961, 4.3 ± 0.9 vs 1.8 ± 0.3 µL (5 min)^−1^ mg^−1^, *p* < 0.001 (Figure 3). Liver insulin clearance was also inhibited by IR blockade (0.64 ± 0.31 vs 0.07 ± 0.01 µL (5 min)^−1^ mg^−1^; *p* = 0.01) (Figure 4).

In group 2 rats, while plasma radiolabeled insulin retention did not differ between HFD and chow-fed rats, HFD increased renal insulin clearance compared with age-matched controls (5.2 ± 0.6 vs 2.8 ± 0.5 µL (5 min)^−1^ mg^−1^; *p* < 0.01). Initial liver insulin clearance was approximately 30% (0.39 ± 0.06 and 0.6 ± 0.10 µL (5 min)^−1^ mg^−1^) less with HFD, but this change was of borderline statistical significance (*p* = 0.056). Skeletal muscle’s initial insulin clearance was unaffected by HFD compared to age-matched chow diet rats (0.070 ± 0.010 vs. 0.078 ± 0.007 µl (5 min)^-1^ mg^-1^, respectively; *p* = 0.7).

## 4. Discussion

Here, we first used the IR specificity of S961 to test whether IRs facilitated the initial rate of whole-body as well as hepatic, renal, and skeletal muscle insulin clearance in vivo. The first step in insulin’s clearance from plasma involves its distribution via the circulation through the plasma compartment. It can then bind to accessible binding sites (e.g., insulin or IGF-1 receptors) on the vascular endothelium or on the liver (whose fenestrated endothelium allows more direct contact of plasma to hepatocytes). In tissues with a continuous endothelium, insulin binding is followed by crossing the vessel wall which separates plasma from the tissue interstitial fluid. In this study, by measuring clearance over a short, 5 min interval using physiologically relevant insulin concentrations, we intentionally sought to assess tissue contributions to these initial steps of insulin clearance. In support of this, in human studies, Sherwin et al. [13] found that a 3-compartment model -with plasma as the compartment that fills immediately, liver (splanchnic) next, and muscle more slowly- adequately described the kinetics of injected insulin’s plasma appearance and clearance. In the rat, Philippe et al. used ^3^H-labeled insulin [14] and found that insulin’s initial volume of distribution (Vd) was greater in the streptozotocin (STZ) diabetic vs control animals. Pre-treating the diabetic rats with a single dose of unlabeled insulin eliminated this difference, suggesting that the initial Vd includes a binding component that was blocked by the unlabeled insulin. Additionally, unlabeled insulin given 6 min after injection of labeled insulin immediately raised the plasma labeled insulin concentration, indicating the competitive displacement of the label from binding sites accessible to plasma. Others have also seen this displacement of insulin from reversible vascular binding sites [11,15] in non-diabetic animals.

The capillary endothelium forms a barrier with a permeability that varies widely across organs. The liver is responsible for the bulk of endogenous insulin clearance, partly through a first-pass effect. This process is likely mediated by IRs on the hepatocyte, given the highly permeable, fenestrated hepatic endothelium. Underscoring the hepatocyte IR’s role (as opposed to the hepatic endothelial cell (EC) IRs) for liver insulin clearance, hepatocyte-specific expression of a dominant-negative carcinoembryonic antigen-related cell adhesion molecule 1 (CEACAM1), which is necessary for liver cell IR and insulin internalization, inhibits insulin clearance, provoking hyperinsulinemia and metabolic insulin resistance [2]. Current data show that S961 decreased liver uptake of labeled insulin by ~85%, an effect greater than we found in muscle or kidney, or previously in the brain [5], confirming the critical role for hepatocyte IRs in hepatic insulin clearance. HFD decreased liver insulin clearance by ~30%, which is comparable to the decline of insulin clearance by perfused livers of rats fed HFD for 3.5 weeks [16].

Muscle, unlike the liver, has a relatively tight, continuous endothelium, which must be crossed for insulin to act on target myocytes [17]. In the kidney, insulin must either cross the glomerular fenestrated capillaries and be taken up from the tubular lumen or cross the capillary in post-glomerular peritubular capillaries [18]. Crossing the endothelium likely poses a rate-limiting barrier to insulin clearance by muscle or kidney and clearly does so in the brain, limited by the blood–brain barrier [5]. Vascular ECs can transport insulin, albumin, and other proteins across the vessel wall [19,20,21]. The demonstrated effect of S961 to decrease insulin clearance from plasma underscores the IR’s importance to whole-body insulin kinetics while its inhibition of liver, muscle, and renal uptake indicates IR involvement in insulin clearance by multiple tissues.

Insulin clearance by the kidney (per gram of tissue) exceeds that of any other organ [18]. Arteriovenous differences in insulin concentration are 30% in men [22]. However, only a small portion of the filtered load appears in urine, the rest is degraded [23]. In the kidney, insulin clearance is complex as insulin may enter the renal cortical cells either by crossing the vascular endothelium of peritubular capillaries or via reabsorption from the tubular lumen after crossing the fenestrated endothelium of the glomerulus. The latter process appears to be mediated by Low density lipoprotein receptor-related protein 2(LRP-2 or megalin), a multi-ligand binding receptor, not IR [24]. Both the short 5 min time-interval of labeled insulin exposure and the observed dramatic impact of S961 to reduce renal tracer uptake suggest that in the current studies, S961 is acting principally on the peritubular capillary. The finding that HFD increases the initial rate of renal insulin clearance may indicate a compensatory role for the kidney to limit peripheral hyperinsulinemia seen with HFD feeding. Renal blood flow increases with HFD feeding as does GFR. The increased renal flow may contribute to the increased insulin clearance seen here in the HFD rats.

The inhibition of muscle insulin clearance by S961 supports the hypothesis that IRs, likely at the microvascular EC, mediate muscle insulin uptake. This is in accord with prior work demonstrating transcellular insulin movement across the EC in skeletal muscle microvasculature [20] and that this process is saturable [7]. By contrast, a recent study tracing transport of fluorescent-tagged insulin across mouse muscle capillaries suggested that IRs were not involved, rather favoring insulin’s passive movement with bulk fluid transport [25]. However, the insensitivity of the fluorescence method employed in those studies necessitated the use of high insulin doses (2–4 U/kg body weight) with resulting plasma insulin concentrations estimated > 10,000 µU/mL. This would far exceed the saturation of IR binding and favor the bulk fluid movement pathway reported. Indeed, recent work using adipose microvascular endothelial cells directly confirms two distinct pathways for endothelial cell insulin uptake with IR-mediated transport at physiological insulin concentrations and fluid-phase transport with pharmacologic insulin concentrations [26].

We previously measured steady-state clearance of endogenous insulin by forearm skeletal muscle in fasting humans (0.0190 µL/5 min/mg tissue, and during a 1 mU/min/kg euglycemic insulin clamp [27]. We reported a significant decline in skeletal muscle insulin clearance during physiologic hyperinsulinemia, indicative of a saturable transport process. Given the significant saturation of clearance with hyperinsulinemia, the involvement of the insulin receptor seemed likely. The current finding that S961 blocks muscle insulin uptake supports that involvement.

The lack of effect of HFD on muscle insulin clearance was perhaps unexpected since insulin-induced muscle microvascular recruitment is blunted after 2 weeks HFD [26]. This suggests that HFD may be acting at a downstream insulin signaling, not on binding per se. Therefore, tissue EC insulin uptake may be affected later than vasorelaxation or not be affected at all. It is also possible that the less abundant perfusion of muscle (~1/20th that of liver, kidney, or brain on a flow/g tissue basis) decreased our ability to detect modest changes in tracer uptake in these short-duration studies.

It is interesting to compare the clearance of insulin by the tissue studied here with known rates of insulin delivery to these tissues as indicated by their respective tissue blood flows. We did not measure tissue-specific blood flow in the current study. However, representative values for barbiturate anesthetized, healthy rats from multiple published studies are available. Table 1 illustrates the relationships between blood flow to each tissue and insulin clearance values. The extraction ratio for insulin is also given. Since all tissues studied received the same arterial concentration of labeled insulin throughout the five min infusion, the tissue extraction ratio can be calculated simply from the tissue blood flow divided by the insulin clearance. We included data on brain insulin clearance which we previously published [5]. The similar extraction ratio (18–20%) by kidney and muscle, despite very different blood flows per gram tissue, underscores the importance of insulin delivery for its clearance. The somewhat lower extraction ratio for the liver may be due to the fact that liver extraction of labeled insulin is competing with the portal rather than the systemic insulin concentration. The former being 2–3-fold higher in concentration, the lower extraction ratio would be expected in a saturable system. Brain insulin clearance appears to be the outlier, the brain having a much more robust blood flow than muscle but a comparable insulin clearance. As a result, its extraction ratio is only ~1/10 that of either muscle, kidney, or likely liver (the latter were corrected for portal insulin concentration).

Clearly, the endothelium of the blood–brain barrier is substantially more restrictive than that of the other tissues studied here. However, S961 lowers insulin’s clearance by all tissues, underscoring the important role of the insulin receptor.

## 5. Conclusions

The endothelial IR rapidly binds insulin and facilitates its clearance by skeletal muscle and kidney. Four weeks of HFD does not affect muscle insulin clearance but may blunt liver clearance and significantly increases renal clearance. The latter may be an adaptation that limits hyperinsulinemia and secondary insulin resistance.

## Figures and Tables

**Table 1:** Tissue-specific blood flow, insulin clearance, and extraction ratio from rats studied under protocol 1 not treated with S961. Data for brain blood flow, clearance, and extraction ratio (ER) are provide for comparison.

Tissue	Blood Flow	Insulin Clearance	Extraction Ratio
	(mL/min/g tissue)	(mL/5 min/g tissue)	(%)
Skeletal muscle	0.06–0.07	0.07	~20%
Kidney	5–6	4.3	~18%
Brain (Cortex)	0.8	0.07	~1.75%
Liver	1.4	0.64	~9.1%

## Data Availability

Primary data are available from the corresponding author upon reasonable request.
